# Investigating Optic Nerve Sheath Diameter in Prone Position Spinal Surgery Patients: A Pilot Study

**DOI:** 10.18502/jovr.v19i3.13863

**Published:** 2024-09-16

**Authors:** Aneesh Rahangdale, Elise Fernandez, Douglas S Weinberg, David Fleischman

**Affiliations:** ^1^Department of Psychiatry, University of Central Florida at HCA Florida Capital Hospital, Tallahassee, FL, USA; ^2^Department of Orthopedics, University of North Carolina, Chapel Hill, NC, USA; ^3^Department of Ophthalmology, University of North Carolina, Chapel Hill, NC, USA; ^4^School of Medicine, University of North Carolina, Chapel Hill, NC, USA

**Keywords:** Cerebrospinal Fluid, Ischemic Optic Neuropathy, Ocular Ultrasound, Optic Nerve Sheath, Prone Position, Spinal Surgery

## Abstract

**Purpose:**

This study aimed to evaluate the effect of intraoperative positioning and ocular immobility on the amount of cerebrospinal fluid around the optic nerve in patients undergoing prone spinal surgery by measuring the optic nerve sheath diameter (ONSD) using ultrasound.

**Methods:**

Consecutive participants (*n* = 15 patients, 30 eyes) were scanned preoperatively, intraoperatively approximately 20 minutes before the end of the surgery, and postoperatively in the post-anesthesia care unit at least 10 min after the completion of the surgery at one academic hospital.

**Results:**

On average, patients who underwent prone spinal surgery had a 21% increase in ONSD intraoperatively, with a positive time-dependent relationship with the overall length of surgery (*P* < 0.001). ONSDs postoperatively returned to baseline and were not significantly different from preoperative measurements.

**Conclusion:**

Our findings suggest pooling and inadequate clearance of perioptic cerebrospinal fluid during prone spinal surgery that improves following termination of the procedure and return of the patient to an upright position.

##  INTRODUCTION

Visual loss after spinal surgery is a rare perioperative complication with an incidence ranging from 0.01% to 2%.^[1,2]^ A study using a large nationwide database in the United States found that out of 2,511,073 spinal surgery cases from 1998 to 2012, 257 patients (1.02/10,000) developed ischemic optic neuropathy (ION).^[3]^ The most common cause of perioperative vision loss (POVL) is ION, with posterior ischemic optic neuropathy (PION) being more common than anterior ischemic optic neuropathy (AION).^[1,4,5]^ PION is a sudden onset optic neuropathy caused by ischemia of the retrobulbar portion of the optic nerve, whereas AION is caused by ischemia of the intraocular portion of the optic nerve.^[2,6]^

Despite the rarity of ION, it is an important perioperative complication because the vision impairment caused by ION is irreversible and often severe and bilateral.^[4]^ Several risk factors are thought to be associated with the development of PION during prone spinal surgeries including intraoperative hypotension, high levels of intraoperative blood loss, and longer operative times.^[4]^ However, prone positioning seems to be an important risk factor since surgeries with patients in these positions are the most commonly affected.^[6,7]^

IONs are pathologies associated with mechanical perfusion. We theorize that significantly increased cerebrospinal fluid (CSF) in the orbital subarachnoid space (OSAS) likely causes external compression of the nerve pial vasculature, especially in the posterior optic nerve where the nerve sheath is less distensible.^[8]^ This accumulation of CSF combined with intraoperative hypotension (caused by operative blood loss, decreased vascular tone from anesthetics, etc.) and the long duration of surgery create conditions for localized infarction of the posterior optic nerve potentially leading to PION.^[9]^

A recently elucidated system termed the “glymphatic system” has been associated with CSF transport and clearance from the retina and optic nerve, as well as the brain.^[10,11]^ It has been suggested as a mechanism for metabolic waste drainage from the central nervous system, and glymphatic dysfunction has been linked to certain neurodegenerative diseases, such as Alzheimer's disease and glaucoma.^[10,12,13,14,15]^ Additionally, rapid eye movement (REM), anesthesia, and body positioning have been suggested as drivers for CSF movement and clearance via the glymphatic system.^[12,13,16,17,18]^ These factors are likely important in proper glymphatic functioning of the optic nerve, therefore, the implications of this study are similarly relevant within this context.

We sought to examine the accumulation of CSF in the perioptic space and its post-operative clearance once the patients were no longer positioned in prone.

**Figure 1:**
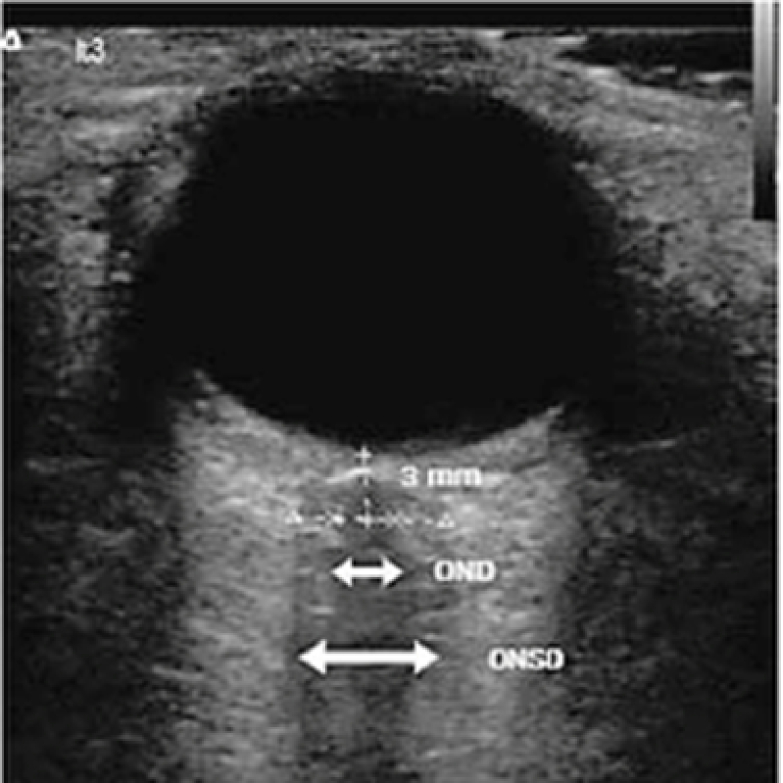
Ultrasound of the eye depicting anatomical landmarks. Optic Nerve Diameter (OND) and Optic Nerve Sheath Diameter (ONSD) were measured 3 mm from the back of the globe in this study.^[19]^.

**Figure 2:**
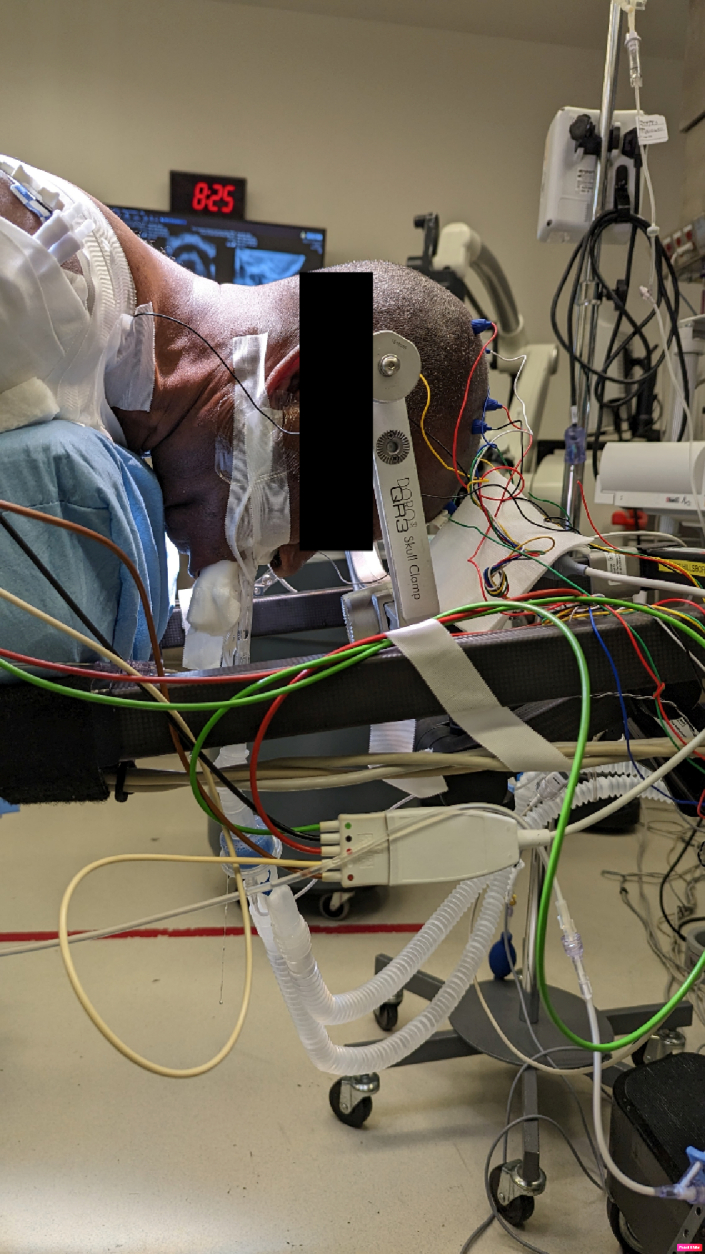
Patients were positioned prone intraoperatively in the operating room on a Jackson spine frame. Per routine protocol, care was taken to pad all bony prominences and avoid any pressure on the face or eyes. Patients were then placed in the reverse Trendelenburg position to aid in surgical exposure and decrease facial venous congestion. They were then prepped and draped in sterile fashion with care taken to avoid any caustic agents near the face or eyes.

**Table 1:** Patient demographics.Patients' age ranged from 44 to 70 years with more White and male patients and had spinal surgeries lasting 5.7 hr on average.


Number of participants	15
Number of eyes	30
Right eye	15
Left eyes	15
Female patients	6
Male patients	9
White patients	8
African American patients	4
Hispanic patients	3
Age range (mean)	44–70 (61)
Average length of prone surgery, hours (SD)	5.7 ± 1.7
	
	
SD, standard deviation	

**Table 2:** Summative results describing how intraoperative ONSD increased but returned to preoperative baseline after the surgery.


**Summative Results, avg (SD)**	**PreOP**	**IntraOP**	**PotstOP**	IntraOp – PreOp*	IntraOp – PostOP*
OND (cm)	0.28 ± 0.06	0.35 ± 0.06	0.27 ± 0.06	0.07 ± 0.04	0.08 ± 0.04
ONSD (cm)	0.53 ± 0.08	0.63 ± 0.06	0.52 ± 0.08	0.10 ± 0.05	0.11 ± 0.05
ONSD – OND ${\;}^{*}$	0.25 ± 0.05	0.28 ± 0.06	0.25 ± 0.05	0.03 ± 0.04	0.03 ± 0.03
	
	
*These indicate the subtraction of values: ONSD minus OND, IntraOP avg minus PreOP avg, IntraOP avg minus PostOP avg. The average ONSD preoperatively was 0.53 cm, which increased to 0.63 cm intraoperatively (*P* < 0.001). Postoperatively, the average ONSD did not show a statistically significant difference from the preoperative measurement. Standard deviations for measurements are written in parentheses. OND, optic nerve diameter; ONSD, optic nerve sheath diameter; PreOP, preoperative measurement; IntraOP, intraoperative measurement; PostOP, postoperative measurement.					

**Figure 3:**
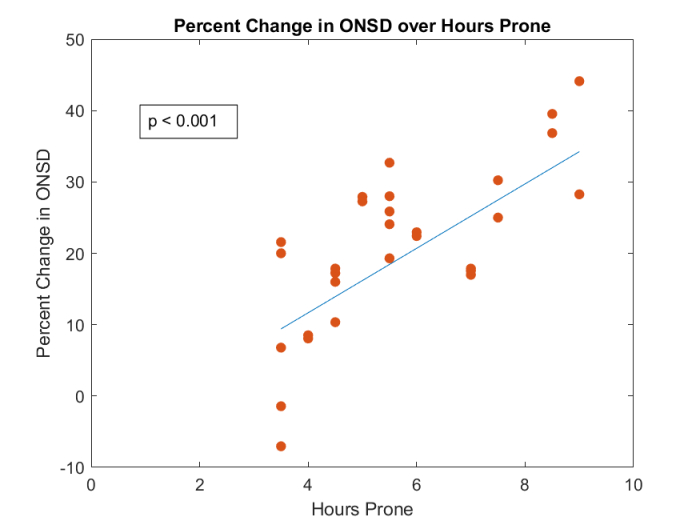
Percent change in ONSD over hours prone depicts a positive correlation between time in the prone position and the percent increase in ONSD (*P* < 0.001). On average, patients undergoing prone spinal surgery had a 21% increase in ONSD intraoperatively. Patients were prone for at least 3.5 hr and on average were prone for 5.7 hr. Notably, one patient prone for 9 hr had a 44% increase in ONSD intraoperatively. Correlation parameters were calculated using method of quasi-least squares. See Supplemental Table 2 for estimated parameters and *P*-values. ONSD, optic nerve sheath diameter.

##  METHODS

This was an observational study that compared optic nerve sheath diameter (ONSD) in patients undergoing spinal surgery before, during, and following surgery in the prone position. An ultrasound (Sonosite M Turbo Portable Ultrasound machine with 6-13 MHz linear ultrasound probe) was used to measure optic nerve diameter (OND) and ONSD pre-, intra-, and post-operatively. An orbital probe was used and adjusted to give a suitable angle for displaying the entry of the optic nerve into the globe. The OND and ONSD were measured at a depth of 3 mm behind the globe [Figure 1]. For each optic nerve, three measurements were recorded in the transverse plane. All ocular scans were performed by the same individual (AR) to account for user variability.

A preoperative measurement was used to establish the patient's baseline. A subsequent measurement was performed at least 3 hours intraoperatively with the patient in the prone surgical position. The intraoperative scan was performed at least 20 minutes before the end of surgery, while the patient was under general anesthesia. Notably, the type of anesthesia could affect intracranial pressure (ICP).^[20]^ Finally, measurements were performed postoperatively in the post-anesthesia care unit (PACU) after the patient was placed in the supine position no longer than 10 minutes after the surgery.

Average OND and ONSD were calculated and compared at each time point using a two-tailed paired *t*-test. The correlation between time in prone position and percent change in ONSD intraoperatively was calculated using generalized estimating equations, specifically, the method of quasi-least squares. Correlation parameters were estimated for time in the prone position, as well as for inter-eye correlation given that for each subject both eyes were enrolled in the study. Statistical analysis was performed in MATLAB using the GEEQBOX toolbox.^[21]^

All subjects gave their informed consent for inclusion before they participated in the study. This study protocol was approved by the UNC Institutional Review Board [IRB and Office of Human Research Ethics, University of North Carolina – Chapel Hill, United States, Study #21-1837] and adhered to the tenets of the Declaration of Helsinki. Subjects were adults over 18 years of age recruited from those undergoing spine surgery at the UNC Department of Orthopedics who consented to be included in this study. Exclusion criteria comprised missing one or both eyes and/or optic nerves, past surgery affecting the optic nerves, optic nerve edema, optic nerve atrophy, inability to keep the eyes in a fixed position for 5 min ± at a time, inability to lie in a supine or prone position, or unwillingness or inability to participate.

##  RESULTS

A total of 15 participants (30 eyes) were recruited for the study. There were more males than females. The demographics of the participants included White, African American, and Hispanic patients. The average age of the patients was 61 ± 8.6 years, and on average patients underwent 5.7 ± 1.7 hours of prone surgery [Table 1]. Patients were prone for at least 3.5 hours.

The average ONSD preoperatively was 0.53 cm, which increased to 0.63 cm intraoperatively [Table 2]. This increase was statistically significant (*P* < 0.001). On average, patients who underwent prone spinal surgery showed a 20.7% increase in ONSD intraoperatively [Supplemental Table 1]. Moreover, there was a statistically significant positive correlation between time in the prone position and the percent increase in ONSD (*P* < 0.001) [Figure 3]. Notably, one patient prone for 9 hr had a 44% increase in ONSD intraoperatively. Postoperatively, the average ONSD was 0.52 cm, which was not significantly different from the preoperative measurement [Table 2]. See Supplemental Table 2 and Supplemental Table 3 for further information on statistical results.

##  DISCUSSION

The results highlight a 20.7% increase (corresponding to 0.1 cm) in ONSD intraoperatively, on average, suggesting pooling and inadequate CSF clearance during prone spinal surgery. It is important to note that the patient positioning as described in the Methods section was not different from that of the surgeon's usual standard protocol and is therefore representative of the majority of prone surgical procedures. Patients with longer periods of time prone during surgery demonstrated larger changes in ONSD, suggesting a time-dependent relationship. We suspect that PION may be at a greater risk of development in situations of increased perioptic CSF volume, iatrogenic anemia, and hypotension.

There were no significant differences between OND and ONSD pre- and post-operatively, suggesting return to normal CSF clearance after the conclusion of surgery, return to independent breathing and supine positioning, and volitional eye movement, consistent with prior studies.^[22,23]^ This also suggests that CSF has not been bottlenecked in these cases, a clinical scenario caused by impaired peri-canalicular flow described by Killer et al.^[24]^ This study also supports previous studies that identified CSF pooling in the perioptic subarachnoid space in an animal model under general anesthesia^[25]^ and Liu and Kahn's perioptic CSF movement studies in a laboratory-based cadaveric *ex vivo* model.^[26]^

Taken in the context of known glymphatic involvement of the optic nerve, perioptic CSF clearance mechanisms are important to elucidate.^[27]^ A lack of proper CSF clearance may impair waste product removal after glymphatic upregulation, which is normally seen during stage 3 sleep, just prior to REM.^[16]^ We suspect that REM during REM sleep (paradoxical sleep) may be an evolutionary mechanism designed to clear CSF from the orbit following glymphatic cleansing during Stage 3 sleep. This may have pathophysiological consequences in long-term eye immobility, such as chronic progressive external ophthalmoplegia, or in conditions with frequent awakenings prior to deep sleep (as in obstructive sleep apnea). The immobility of globes accentuated in this study suggests that eye movement may be mechanistically involved in the process of CSF clearance from the perioptic space.

Limitations of this study include generalizability due to the middle-aged patient population (no patients under age 44) and relatively long surgery length (no surgeries under 3.5 hours). Moreover, intraocular and ICP, episodes of intraoperative hypotension, intraoperative mean arterial pressure, pulse rate, and variability in total blood loss were not factored into our individual data points and may have affected ONSD. Given the potential effects of these confounders on ONSD, it is difficult to deduce from this study the role that eye movement plays in the pathophysiology of CSF pooling and clearance in the perioptic space. Further research may elucidate whether differences in the aforementioned variables affect the findings. Lastly, the sample size was 30 eyes of 15 patients, but we believe that this will at least provide a direction for future investigations with a greater number of subjects.

Future studies measuring OND and ONSD in patients with various positionings and in the presence or absence of eye movements are needed to better understand the effects of these variables on CSF pooling around the optic nerve. Potential ways to accomplish this include measuring the OND and ONSD in patients who spend long periods of time in prone position following retinal surgery, or in healthy patients instructed to rest in various positions for shorter periods of time. Additionally, further investigation looking at intraoperative blood pressure and ICP in spinal surgery patients is warranted to rule out the effects of these on ONSD. The findings of this study and future studies on CSF movement in the perioptic space can provide a roadmap for potential intraoperative countermeasures to protect against PION, as well as a better understanding of other diseases that affect the optic nerve.

### Acknowledgements 

The authors are thankful to the hospital staff that facilitated patient care and to the patients who participated in this study.

### Financial Support and Sponsorship

None.

### Conflicts of Interest

None.
